# Novel *SSR4* gene splice variant leads to congenital disorder of glycosylation, type Iy

**DOI:** 10.3389/fped.2025.1651524

**Published:** 2025-10-24

**Authors:** Ning Li, Chen Chen

**Affiliations:** ^1^ Pediatric Department, Liuyang People Hospital, Changsha, China; ^2^ Children’s Medical Center, Xiangya Hospital, Central South University, Changsha, China

**Keywords:** *SSR4*, congenital disorder of glycosylation, whole exome sequencing, minigene, transcript

## Abstract

**Background:**

Congenital disorders of glycosylation (CDG) are a group of multi-systemic genetic disorders. Over 100 monogenic human diseases were known related with defects in glycosylation process. Defects of *SSR4* gene lead to a rare X linked pattern of CDG which has been rarely reported.

**Method:**

We reported a Chinese boy with developmental delay, microcephaly, and epileptic seizures. Whole exome sequencing and Sanger sequencing were performed in the family.

**Result:**

A novel maternal splice variant c.351+1del in *SSR4* gene was identified by trio-exome sequencing, and confirmed by Sanger sequencing. The functional effect of the variant was further investigated by minigene. The minigene results showed three abnormal splice forms: (1) 1 bp deletion in 3′ end of exon 4; (2) 42 bp deletion in 3′ end of exon 4; (3) skipping of exon 4. All three forms resulted in truncated proteins. c.351+1del in *SSR4* gene causes congenital disorder of glycosylation, type Iy, consisted with the proband's phenotype. Up to date, all of the pathogenic *SSR4* gene variants were null variants. The most variants were reported in exon 4. Patients (within or between families) carrying the same variants exhibited phenotypic heterogeneity.

**Conclusion:**

The current study expanded the pathogenic variant spectrum of *SSR4* gene and revealed the impact of c.351+1del on *SSR4* splicing. Standardizing the transcript and naming conventions of variants were crucial for the study of *SSR4* genotypes and phenotypes.

## 1 Introduction

N-linked glycosylation is a post-translational modification essential for the folding, stability, and other cellular functions of membrane proteins (1, 2). In recent years, literatures had reported alterations in the N-glycans of membrane proteins are associated with various pathological conditions (3, 4). Signal sequence receptor protein 4 (*SSR4*, OMIM 300090) on chromosome Xq28, encodes a subunit of a transmembrane protein complex involved in the transport of proteins across the endoplasmic reticulum membrane, enhancing the efficiency of N-linked glycosylation (5). Hemizygous variants in the *SSR4* gene cause congenital disorder of glycosylation, type Iy (CDG1Y, OMIM 300934), also called SSR4-CDG. SSR4-CDG is characterized by developmental delay, speech delay, impaired intellectual development, muscular hypotonia, microcephaly, seizures and distinctive facial features (5). Extended phenotype has cardiomyopathy (6) and connective tissue disorder (redundant skin, joint laxity, blue sclerae, and vascular tortuosity) (7). Up to date, more than 20 patients with loss-of-function variants were reported with CDG1Y, including total gene deletions (6 patients), nonsense (5 patients), frameshift (7 patients) and splice variants (5 patients) (5–13). However, the relationship between *SSR4* genotype and phenotype was not yet clear.

We reported a Chinese boy with a maternal splice variant of *SSR4* gene and verified the impact on mRNA coding. To conduct accurate analysis of genotypes, we summarized the characteristics of the *SSR4* genotypes and provided theoretical foundation for the study of the genotype and phenotype of the *SSR4*.

## 2 Materials and methods

### 2.1 Clinical course

The proband, a 4-year-old boy (G1P1), was delivered naturally with a birth weight of 2.7 kg. He underwent developmental delay, microcephaly, and epileptic seizures twice before. He was admitted to the Pediatric of the People's Hospital of Liuyang due to epileptic seizure accompanied by high fever one day ago. He had a high fever with unknown reason (up to 39℃) before seizure. The seizure type was tonic-clonic seizure, characterized by unresponsiveness, eyes rolling up, dark complexion, trembling limbs, and lasting for about 2 min. Further examination after admission revealed swollen tonsils (Ⅰ°) and coarse breath sounds in both lungs. Routine electrolyte test results suggest hypokalemia (potassium, 3.14 mmol/L). No obvious brain structural abnormalities were found on cranial MRI. Urine organic acid test results were normal. Blood amino acid and acylcarnitine profile analysis showed a slight decrease in glutamine (Gln) level (0.89, normal range from 1.00 to 55.00). Facial features included deep set eyes, large ears and mouth. The child's general developmental level was equivalent to 30-month-old according to the Gesell Developmental Diagnostic Scale, with a Developmental Quotient (DQ) of 54, indicating moderate developmental delay. His parents were healthy, non-consanguineous, and had no family history of genetic diseases. After adequate genetic counseling, we performed trios whole exome sequencing (WES) on the proband and his parents.

### 2.2 Whole exome sequencing

Genomic DNA were extracted from peripheral blood samples using Qiagen DNA Blood Midi/Mini kit (Qiagen GmbH, Hilden, Germany). 50 ng genomic DNA was interrupted to approximately 250 bp around by fragmentation enzymes. The DNA fragments were then end repaired, and the 3′end was added one A base. The DNA fragments were ligated with barcoded sequencing adaptors and collected by XP beads. The DNA fragments were hybridized and captured by probe named Nano WES V2 (Berry Genomics, Beijing, China). The products were eluted and collected, and subjected to PCR amplification and the purification. The libraries were quantified by qPCR and sequenced by Novaseq6000 platform (Illumina, San Diego, USA) with 150 bp pair-end sequencing mode.

The raw sequencing reads were aligned to the human reference genome (GRCh38) using Burrows Wheeler Aligner (14) tool and PCR duplicates were removed by using Picard v1.57 (http://picard.sourceforge.net/). Verita Trekker® Variants Detection System by Berry Genomics and GATK (https://software.broadinstitute.org/gatk/) were employed for variant calling. Variant annotation and interpretation were conducted by ANNOVAR (15) and the Enliven® Variants Annotation Interpretation System authorized by Berry Genomics. Annotation databases mainly included gnomAD (http://gnomad.broadinstitute.org/), EXAC database (https://gnomad.broadinstitute.org/), the 1,000 Genome Project (http://browser.1000genomes.org), Berry inhouse population database, dbSNP (http://www.ncbi.nlm.nih.gov/snp), SIFT (http://sift.jcvi.org), FATHMM (http://fathmm.biocompute.org.uk), MutationAssessor (http://mutationassessor.org), CADD (http://cadd.gs.washington.edu), SPIDEX (16), spliceAI (17), FF (https://www.fruitfly.org/seq_tools/splice.html), OMIM (http://www.omim.org), ClinVar (http://www.ncbi.nlm.nih.gov/clinvar), HGMD (http://www.hgmd.org), HPO (https://hpo.jax.org/app/) etc. The variants were classified to five categories (pathogenic, likely pathogenic, uncertain significance, likely benign and benign) according to the American College of Medical Genetics and Genomics (ACMG) guidelines for interpretation of genetic variants (18).

### 2.3 Sanger sequencing

The candidate variant was verified by Sanger sequencing. The primers (Forward: CAGAAGGTGACCCTGCCTTT; Reverse: AAACAGAGGCGGGATGATGG) were designed by Primer 5 software. DNA fragment containing the candidate variant was amplified by polymerase chain reaction: initial denaturation at 95°C for 5 min, followed by 34 cycles at 95°C for 30 s, 58°C for 30 s and 72°C for 20 s and hold at 72°C for 10 min. PCR product was purified and sequenced using an ABI 3730XL DNA Analyzer with the BigDye™ Terminator Cycle Sequencing Kit (Applied Biosystems, Foster, CA, USA). The results of Sanger sequencing were analyzed by Chromas software according to NM_006280.

### 2.4 *In vitro* minigene assays

We conducted *in vitro* minigene assays to verify the impact of candidate variant. To enhance the reliability of the experimental results, we transfected two types of recombinant plasmids into two types of tool cells respectively.

Using normal human gDNA as a template, nested PCRs were carried out by Nested primer 1 and 2. Then using nested PCR products as template to generate the wide and mutant *SSR4* fragments of pcDNA3.1 and pcMINI-C. Pair of pcDNA3.1-SSR4-KpnI-F and pcDNA3.1-SSR4-EcoRI-R were used to generate fragment of pcDNA3.1-SSR4-wt. Using the mixture of PCR reaction products (pcDNA3.1-SSR4-KpnI-F and SSR4-mut-R, SSR4-mut-F and pcDNA3.1-SSR4-EcoRI-R) as template to generate fragment of pcDNA3.1-SSR4-mut by pair of pcDNA3.1-SSR4-KpnI-F and pcDNA3.1-SSR4-EcoRI-R. Pair of pcMINI-C-SSR4-KpnI-F and pcMINI-C-SSR4-EcoRI-R were used to generate fragment of pcMINI-C-SSR4-wt. Using the mixture of PCR reaction products (pcMINI-C-SSR4-KpnI-F and SSR4-mut-R, SSR4-mut-F and pcMINI-C-SSR4-EcoRI-R) as template to generate fragment of pcMINI-C-SSR4-mut by pair of pcMINI-C-SSR4-KpnI-F and pcMINI-C-SSR4-EcoRI-R. All of primers (Table 1) were designed by Primer5 software.

**Table 1:** Sequences and purposes of the primers used in minigene assay.

Purpose	Primer name	Primer sequence (5′-3′)
Generate nested PCR products	Nested primer 1-F	GGTTCAAGCGATTCTCCTCT
	Nested primer 2-F	AGTCAACAGGGTTCCTATGC
	Nested primer 2-R	CAGCTCAGGGGAGTACAGGT
	Nested primer 1-R	TCAGGCCTGGATGTGGCTCT
Generate pcDNA3.1-SSR4-wt and mutant fragments	pcDNA3.1-SSR4-KpnI-F	GCTTGGTACCATGAACATGGCTCTCTATGCTGA
	pcDNA3.1-SSR4-EcoRI-R	TGCAGAATTCCCGATGGTCCACGCTGACTG
	SSR4-mut-F	AGCCTCCTCAGGAAGTGAGGACTCCTGTAG
	SSR4-mut-R	CTACAGGAGTCCTCACTTCCTGAGGAGGCT
Generate pcMINI-C-SSR4-wt and mutant fragments	pcMINI-C-SSR4-KpnI-F	GGTAGGTACCTGAGGGGCCAATGGTTCCCT
	pcMINI-C-SSR4-EcoRI-R	TGCAGAATTCCCGATGGTCCACGCTGACTG
	SSR4-mut-F	AGCCTCCTCAGGAAGTGAGGACTCCTGTAG
	SSR4-mut-R	CTACAGGAGTCCTCACTTCCTGAGGAGGCT
Get cDNA fragments of pcDNA3.1	pcDNA3.1-F	CTAGAGAACCCACTGCTTAC
	pcDNA3.1-R	TAGAAGGCACAGTCGAGG
Get cDNA fragments of pcMINI	pcMINI-C-F	ACTTAAGCTTATGAGTGGGCTTTGGGGTGGCCGGTT
	pcMINI-C-R	TAGAAGGCACAGTCGAGG

The wild and mutant fragments were constructed in pcDNA3.1 and pcMINI-C plasmids (Figures 2A,B) and confirmed by Sanger sequencing. The vector constructions were supplied in the Supplementary Figure. The primers sequences used in vector constructions were show in Table 1. The recombinant plasmids were transfected into HEK293T and HeLa cells separately using Hieff Trans™ Liposomal Transfection Reagent (YEASEN, Shanghai, China). Total RNA was extracted from cells cultured for 48 h with Trizol Reagent (TaKaRa, Kusatsu, Japan) and reverse-transcribed with the Superscript III reverse transcriptase (HifairTM 1st Strand cDNA Synthesis SuperMix for qPCR) (YEASEN, Shanghai, China). PCRs were performed by primers (pcDNA3.1-F and pcDNA3.1-R; pcMINI-C-F and pcMINI-C-R) in the target region of cDNA. The PCR products were analyzed on 1% agarose gel electrophoresis and using an ABI 3730XL DNA Analyzer with the BigDye™ Terminator Cycle Sequencing Kit (Applied Biosystems, Foster, CA, USA). Compare the sequencing results of wild and mutant-type in the cells and analysis the splice form of the mutant type.

### 2.5 The three-dimensional (3D) structure

The three-dimensional (3D) structure of SSR4 was analyzed by PyMOL (The PyMOL Molecular Graphics System, Version 2.0 Schrödinger, LLC), using AF-P51571-F1-model_v4 as the template.

### 2.6 Analysis of the *SSR4* genotype

Using “*SSR4* gene”, “SSR4-CDG” and “congenital disorder of glycosylation, type Iy” as the key words, conduct a search in the PUBMED database. Using “*SSR4*” as the key word to search the transcripts (Homo sapiens) in NCBI database. According to Human Genome Variation Society (HGVS) nomenclature rules (https://hgvs-nomenclature.org/), we checked and corrected the variants reported. We drew the gene structure of *SSR4* based on the transcript structure provided by ProteinPaint (https://proteinpaint.stjude.org/) and performed localization and analysis of the *SSR4* SNVs and indels.

## 3 Results

### 3.1 Results of WES

**Figure 1:**
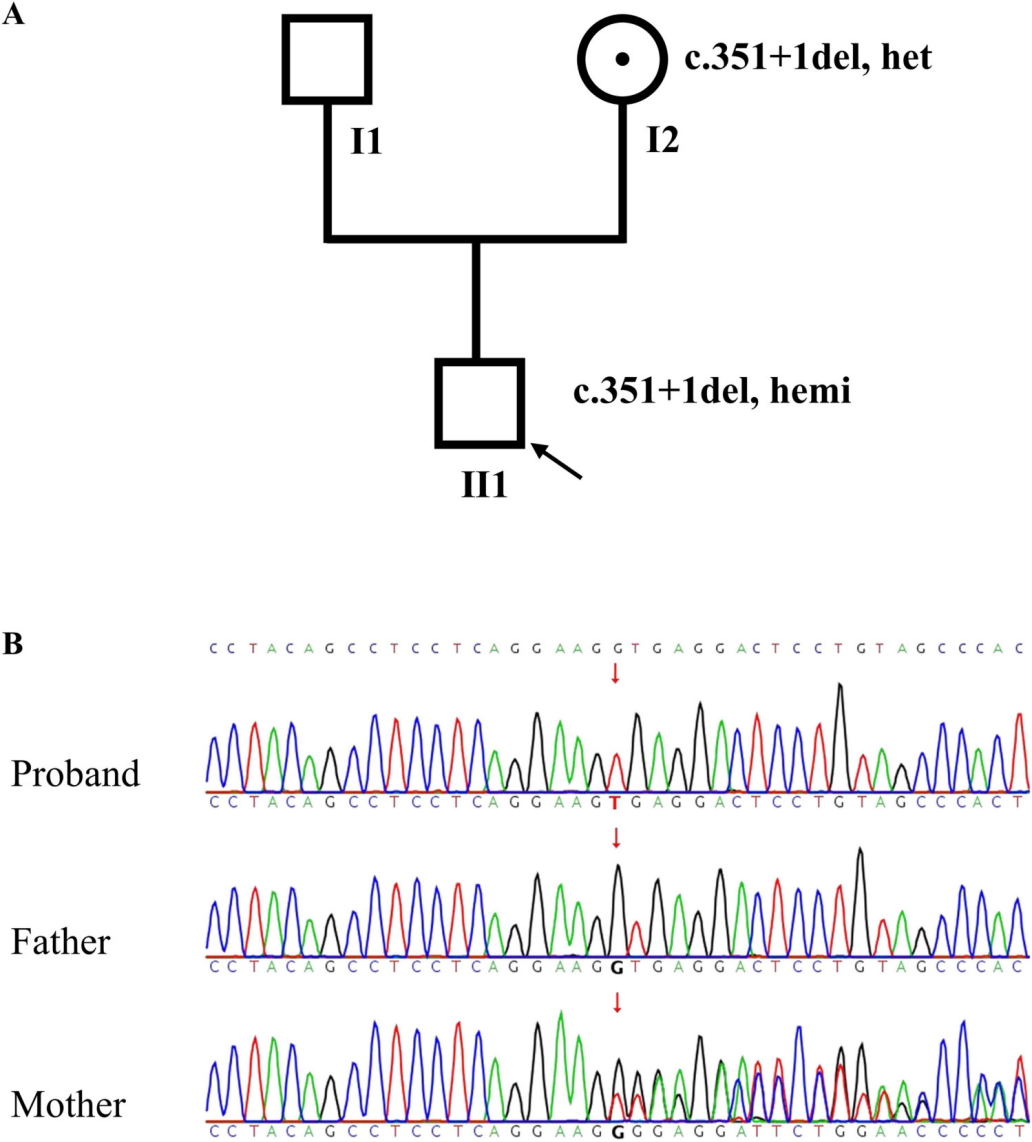
Pedigree and *SSR4* gene sequencing results. **(A)** The pedigree of the family; **(B)** Sanger sequence chromatogram of *SSR4* gene. The results showed that c.351+1del in *SSR4* was hemizygous in the proband (Ⅱ1) and heterozygote in the mother (Ⅰ2).

A novel hemizygous splice variant NM_006280: c.351+1del in *SSR4* gene (OMIM 300090) was identified in the proband by WES. Both of WES and sanger sequencing confirmed it inherited from the mother (Figure 1). c.351+1del was in the intron 4 of *SSR4* gene. It was a splice donor variant, predicted to undergo nonsense-mediated mRNA decay (NMD)(PVS1) (19). Multiple software programs predict the variant impact the splicing of *SSR4* (spliceAI: donor loss 1.00, donor gain 0.98; FF: donor loss 0.99). It wasn't reported in HGMD (https://www.hgmd.cf.ac.uk/ac/index.php) and ClinVar (https://www.ncbi.nlm.nih.gov/clinvar/) databases previously. The frequency of the variant was not found in gnomAD, EXAC, 1000G and Berry inhouse database (PM2_Supporting). c.351+1del was classified as “likely pathogenic” (PVS1+PM2_Supporting) according to ACMG guidelines. *SSR4* gene was related with Congenital disorder of glycosylation, type Iy (CDG1Y, OMIM 300934). Developmental delay, microcephaly, and epileptic seizures were consisted with the clinical phenotype of CDG1Y. The normal mother carried the heterozygote variant c.351+1del, while the proband carried the hemizygous variant. The co-segregation pattern of the family was consistent with X-linked recessive inheritance.

### 3.2 Results of minigene splicing assay (*SSR4* gene c.351+1del)

**Figure 2:**
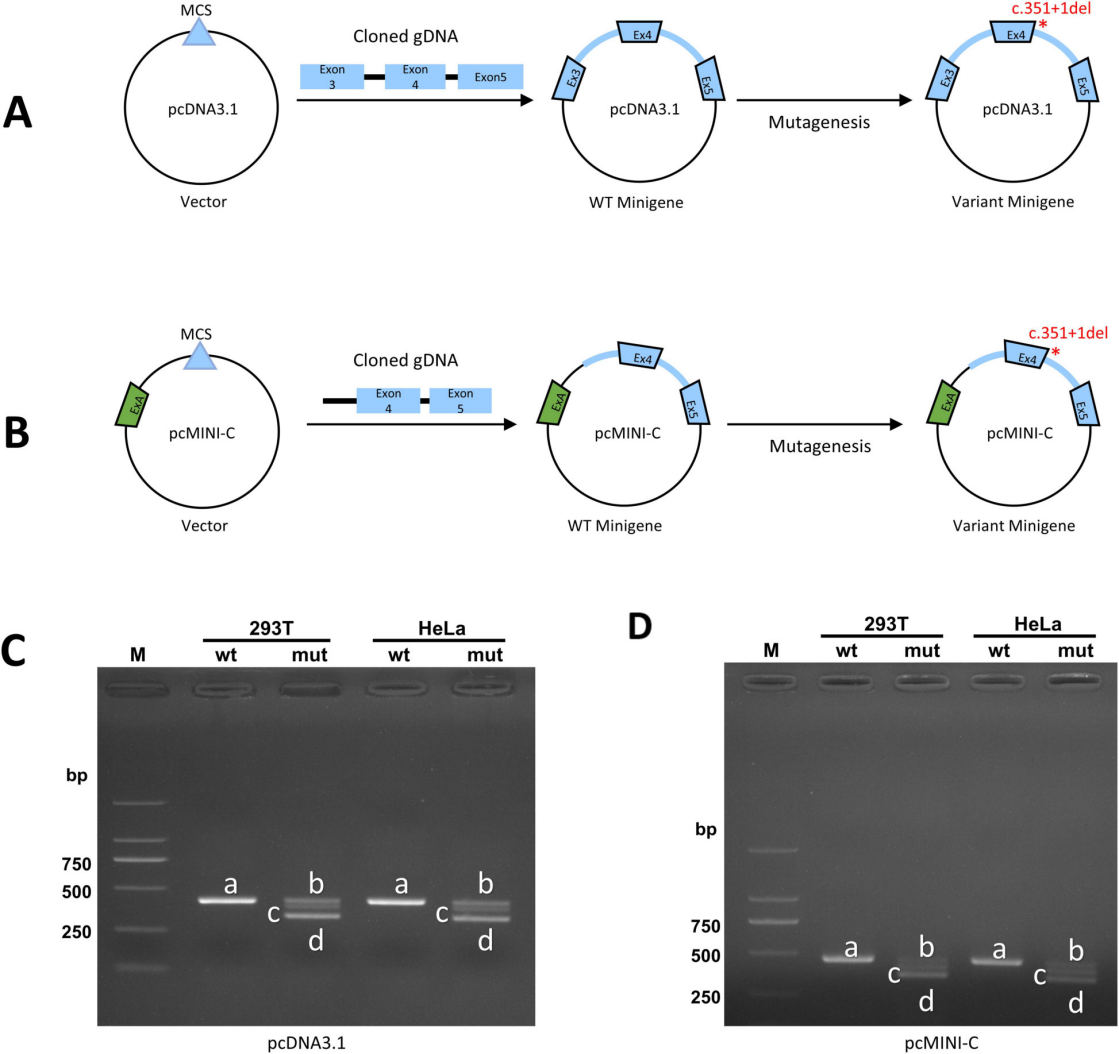
The *in vitro* minigene assay of pcDNA3.1 and pcMINI-C. **(A)** Strategies for plasmid pcDNA3.1 construction; **(B)** Strategies for plasmid pcMINI-C construction; **(C,D)** The Gel electrophoresis of RT-PCR products displayed a single band **(a)** from the wild type (wt) and three different bands **(b–d)** in the mutant type (mut).

**Figure 3:**
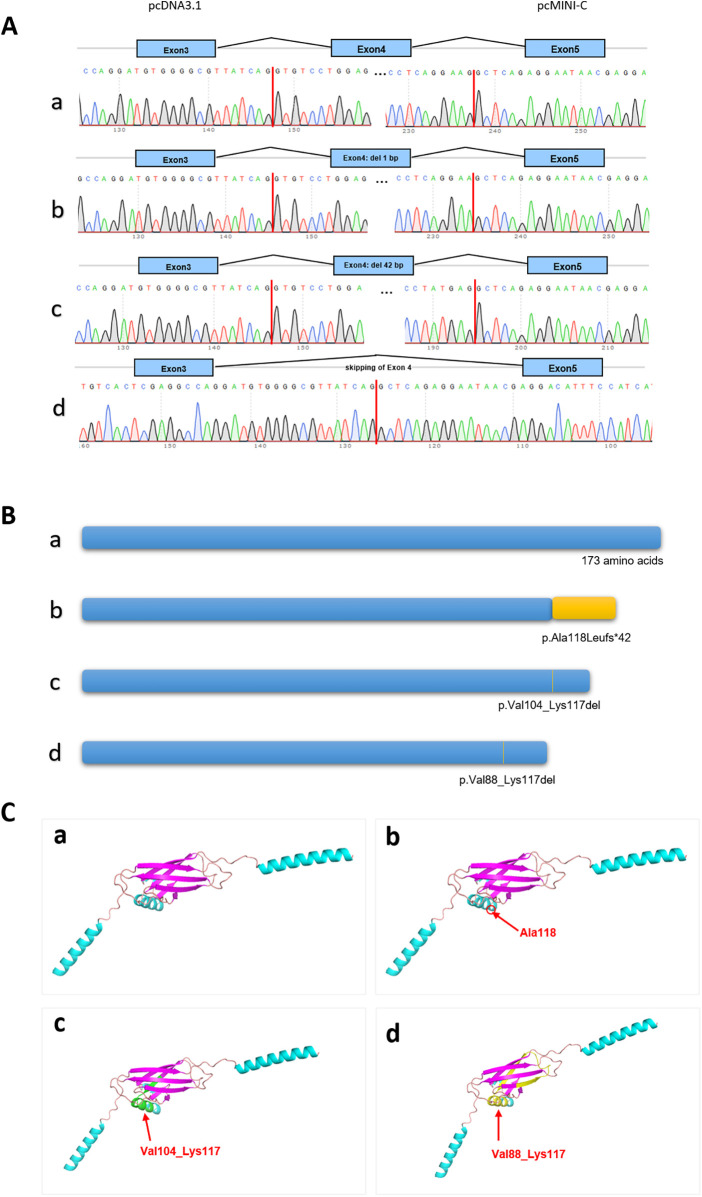
The results of minigene assay of *SSR4* gene c.351+1del. **(A)** The cDNA Sanger sequencing of RT-PCR products in pcDNA3.1 was consistent in pcMINI-C. Band a led to a shorter transcript with 1 bp deletion of exon 4; Band b led to a shorter transcript with 42 bp deletion of exon 4; Band c led to a shorter transcript with skipping of exon 4; **(B)** Schematic diagram of three types of protein length. **(C)** The positions of three different variants on the three-dimensional protein structure diagram.

The functional effect of the variant was investigated by *in vitro* minigene assay. The minigene results of two systems were consisten. There were three abnormal splice forms: (1) 1 bp deletion in 3′ end of exon 4 (c.351del, p.Ala118Leufs*42); (2) 42 bp deletion in 3′ end of exon 5 (c.310_351del, p.Val104_Lys117del); (3) skipping of exon 4 (c.262_351del, p.Val88_Lys117del) (Figures 2, 3). All three forms resulted in the production of truncated proteins.

### 3.3 Genotype of *SSR4*

**Figure 4:**
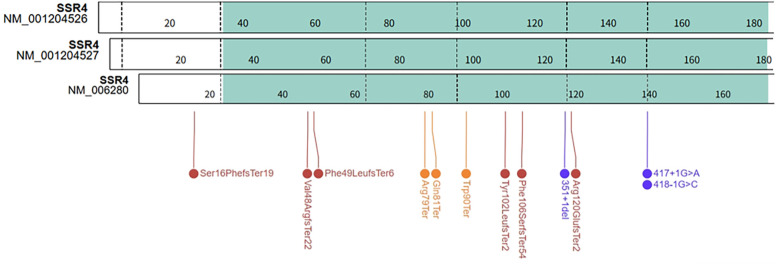
*SSR4* gene transcripts and distribution of reported variants in *SSR4* gene.

There were three transcripts of *SSR4* gene in the NCBI database: NM_006280 (MANE select), NM_001204526 and NM_001204527 (Figure 4). According to the MANE transcript and the HGVS Nomenclature, we standardized the variants of 16 previously reported SSR4-CDG patients and patient in this work (see Supplementary Table). The distribution of variants on the *SSR4* gene (MANE transcript) was summarized in Figure 4. All of the variants distributed in the coding exons and canonical splice sites of NM_006280. The most cases and variants were reported in exon 4 (3 variants in 4 patients), including the recurrent variant NM_006280: c.269G>A (p.Trp90Ter) reported in 2 unrelated patients [NM_006280: c.269G>A (p.Trp90Ter) equivalent to NM_001204527:c.302G>A (p.Trp101Ter)]. While no variant reported in exon 1 of NM_001204526 or NM_001204527.

### 3.4 Follow-up

After six months of language rehabilitation training at our hospital, the child underwent the Gesell Developmental Schedules assessment at the age of four years and ten months. The results indicated that his general developmental level was equivalent to 35-month-old, with a DQ of 56, falling within the range of mild developmental delay. The child later received acupuncture and massage rehabilitation treatment at another hospital. At the age of five years and three months, he was assessed again using the Gesell Developmental Schedules, which showed his general developmental level to be equivalent to 39-month-old, with a DQ of 57, still indicating mild developmental delay.

## 4 Discussion and conclusions

Glycosylation can regulate enzyme activity or its interaction with other proteins to play roles in various cellular processes, including cell recognition, cell signaling, resistance to proteases, adhesion, migration, and host defense mechanisms (20). An important process required for N-glycosylation is the co-translational translocation of proteins from the cytoplasm to the endoplasmic reticulum, which is facilitated by the translocon-associated protein (TRAP) complex. The transmembrane subunit SSR4 is a member of the signal sequence receptor (SSR) family in the structure of the translocon complex (5, 21). When *SSR4* is defective, the addition of glycan precursors to proteins in the endoplasmic reticulum is impaired, potentially leading to disordered N-linked glycosylation of many proteins and the complete loss of glycans (7, 9, 22).

SSR4-CDG is a multisystem disorder characterized by neurological phenotypes (9, 23). Unlike most autosomal recessive CDG disorders, *SSR4* is located on the X chromosome and the pathogenic variants maybe *de novo* or maternal inheritance (5). In our work, the mother was a heterozygote of *SSR4* gene c.351+1del without typical clinical symptoms. Carbohydrate-deficient transferrin (CDT) is a reliable and cost-effective screening method for identifying CDG cases (24). However, it cannot pinpoint specific genetic defects. Previously reported cases of SSR4-CDG had mostly shown Type I transferrin (Tf) patterns (5, 9, 10). Due to the lack of specific clinical features associated with CDG-related disorders, the initial presentation is often global developmental delay (GDD). Most CDG cases were identified through genetic testing and subsequently confirmed CDT. As seen in our work, patients often refuse CDT testing after receiving the positive genetic results. This highlights the importance of considering SSR4-CDG as a potential diagnosis in multisystem disorders with predominant neurological manifestations and conducting genetic testing.

Regarding the clinical phenotype of SSR4-CDG patients, three previous publications have organized and presented cohort samples (5, 9, 10). Our study referenced previously reported cases and standardized the nomenclature of SNVs and indels. After standardizing the variants, we found that two variants were recurrent in two unrelated families: c.269G>A, (p.Trp90Ter) in exon 4 and c.417+1G>A in intron 5. The study by Johnsen C et al (5) showed that P14 and P22 were found to carry c.269G>A, p.(Trp90Ter), exhibiting the same core phenotypes (developmental delay, intellectual disability, muscular hypotonia and abnormal facial features), different the other phenotypes (feeding difficulties, connective tissue, gastrointestinal Symptoms, skeletal abnormalities, behavioral issues). The study by Wang et al (10) showed that c.269G>A leaded to mRNA expression of the *SSR4* gene approaching zero. The study by Johnsen C et al (5) that P6 and P19 carry c.417+1G>A, presenting with a consistent core phenotype but differing in other phenotypic features. c.417+1G>A were predicted might result in the skipping of exon 5. Similar to many other types of CDG, clinical phenotyping revealed heterogeneity among different individuals (25).

Our study conducted minigene to clarify the impact of splice variants on mRNA encoding. The shortest transcript products, as shown in Figures 2C,D, result from the skipping of exon 4, leading to the deletion of amino acids within the reading frame. In the agarose gel electrophoresis results (Figures 2C,D), this product was found to have the highest expression level. All indications suggest that the integrity of the protein encoded by the *SSR4* gene is crucial for its function. Current, the structure of the protein encoded by *SSR4* and the function of the region encoded by exon 4 are not well understood and require further investigation. No variations have been reported in the last exon, and its pathogenicity requires further observation.

In conclusion, the current study expanded the pathogenic variant spectrum of *SSR4* gene and revealed the impact of c.351+1del on *SSR4* splicing. Standardizing the transcript and naming conventions of variants were crucial for the correct study of *SSR4* genotypes and phenotypes.

## Data Availability

The raw data supporting the conclusions of this article will be made available by the authors, without undue reservation.

## References

[1] Huang Z Lai PF Cocker ATH Haslam SM Dell A Brady HJM Roles of N-linked glycosylation and glycan-binding proteins in placentation: trophoblast infiltration, immunomodulation, angiogenesis, and pathophysiology. Biochem Soc Trans. (2023) 51(2):639–53. 10.1042/BST20221406 36929183 PMC10212547

[2] Esmail S Manolson MF. Advances in understanding N-glycosylation structure, function, and regulation in health and disease. Eur J Cell Biol. (2021) 100(7–8):151186. 10.1016/j.ejcb.2021.151186 34839178

[3] Conroy LR Hawkinson TR Young LEA Gentry MS Sun RC. Emerging roles of N-linked glycosylation in brain physiology and disorders. Trends Endocrinol Metab. (2021) 32(12):980–93. 10.1016/j.tem.2021.09.006 34756776 PMC8589112

[4] Cawley NX Lyons AT Abebe D Luke R Yerger J Telese R Complex N-linked glycosylation: a potential modifier of Niemann-Pick disease, type C1 pathology. Int J Mol Sci. (2022) 23(9):5082. 10.3390/ijms23095082 35563467 PMC9103943

[5] Johnsen C Tabatadze N Radenkovic S Botzo G Kuschel B Melikishvili G SSR4-CDG, an ultra-rare X-linked congenital disorder of glycosylation affecting the TRAP complex: review of 22 affected individuals including the first adult patient. Mol Genet Metab. (2024) 142(3):108477. 10.1016/j.ymgme.2024.108477 38805916

[6] Zemet R Hope KD Edmondson AC Shah R Patino M Yesso AM Cardiomyopathy, an uncommon phenotype of congenital disorders of glycosylation: recommendations for baseline screening and follow-up evaluation. Mol Genet Metab. (2024) 142(4):108513. 10.1016/j.ymgme.2024.108513 38917675 PMC11296892

[7] Castiglioni C Feillet F Barnerias C Wiedemann A Muchart J Cortes F Expanding the phenotype of X-linked SSR4-CDG: connective tissue implications. Hum Mutat. (2021) 42(2):142–9. 10.1002/humu.24151 33300232

[8] Wu R Tang W Qiu K Li X He Z. [Analysis of SSR4 gene variant in a child with congenital glycosylation type 1y in conjunct with congenital dysplasia of external auditory canal]. Zhonghua Yi Xue Yi Chuan Xue Za Zhi. (2022) 39(7):727–30. Chinese. 10.3760/cma.j.cn511374-20210205-0011 35810430

[9] Ng BG Raymond K Kircher M Buckingham KJ Wood T Shendure J Expanding the molecular and clinical phenotype of SSR4-CDG. Hum Mutat. (2015) 36(11):1048–51. 10.1002/humu.22856 26264460 PMC4604052

[10] Wang J Gou X Wang X Zhang J Zhao N Wang X. Case report: the novel hemizygous mutation in the SSR4 gene caused congenital disorder of glycosylation type Iy: a case study and literature review. Front Genet. (2022) 13:955732. 10.3389/fgene.2022.955732 36386804 PMC9643473

[11] Sun W Jin X Zhu X. A novel SSR4 variant associated with congenital disorder of glycosylation: a case report and related analysis. Front Genet. (2024) 15:1402883. 10.3389/fgene.2024.1402883 39086474 PMC11288868

[12] Weng L Deng Q Chen X Wang K Shao J. A case of congenital disorder of glycosylation due to SSR4 gene deletion. Zhonghua Yi Xue Yi Chuan Xue Za Zhi. (2023) 40(3):364–7. 10.3760/cma.j.cn511374-20210918-00762 36854416

[13] Losfeld ME Ng BG Kircher M Buckingham KJ Turner EH Eroshkin A A new congenital disorder of glycosylation caused by a mutation in SSR4, the signal sequence receptor 4 protein of the TRAP complex. Hum Mol Genet. (2014) 23(6):1602–5. 10.1093/hmg/ddt550 24218363 PMC3929095

[14] Li H Durbin R. Fast and accurate short read alignment with Burrows-Wheeler transform. Bioinformatics (Oxford, England). (2009) 25(14):1754–60. 10.1093/bioinformatics/btp324 19451168 PMC2705234

[15] Wang K Li M Hakonarson H. ANNOVAR: functional annotation of genetic variants from high-throughput sequencing data. Nucleic Acids Res. (2010) 38(16):e164. 10.1093/nar/gkq603 20601685 PMC2938201

[16] Xiong HY Alipanahi B Lee LJ Bretschneider H Merico D Yuen RK RNA splicing. The human splicing code reveals new insights into the genetic determinants of disease. Science. (2015) 347(6218):1254806. 10.1126/science.1254806 25525159 PMC4362528

[17] Jaganathan K Kyriazopoulou Panagiotopoulou S McRae JF Darbandi SF Knowles D Li YI Predicting splicing from primary sequence with deep learning. Cell. (2019) 176(3):535–48.e24. 10.1016/j.cell.2018.12.015 30661751

[18] Richards S Aziz N Bale S Bick D Das S Gastier-Foster J Standards and guidelines for the interpretation of sequence variants: a joint consensus recommendation of the American college of medical genetics and genomics and the association for molecular pathology. Genet Med. (2015) 17(5):405–24. 10.1038/gim.2015.30 25741868 PMC4544753

[19] Abou Tayoun AN Pesaran T DiStefano MT Oza A Rehm HL Biesecker LG Recommendations for interpreting the loss of function PVS1 ACMG/AMP variant criterion. Hum Mutat. (2018) 39(11):1517–24. 10.1002/humu.23626 30192042 PMC6185798

[20] He M Zhou X Wang X. Glycosylation: mechanisms, biological functions and clinical implications. Signal Transduct Target Ther. (2024) 9(1):194. 10.1038/s41392-024-01886-1 39098853 PMC11298558

[21] Huang Y Xu X Arvan P Liu M. Deficient endoplasmic reticulum translocon-associated protein complex limits the biosynthesis of proinsulin and insulin. FASEB J. (2021) 35(5):e21515. 10.1096/fj.202002774R 33811688 PMC8106808

[22] Ng BG Lourenco CM Losfeld ME Buckingham KJ Kircher M Nickerson DA Mutations in the translocon-associated protein complex subunit SSR3 cause a novel congenital disorder of glycosylation. J Inherit Metab Dis. (2019) 42(5):993–7. 10.1002/jimd.12091 30945312 PMC6739144

[23] Francisco R Brasil S Poejo J Jaeken J Pascoal C Videira PA Congenital disorders of glycosylation (CDG): state of the art in 2022. Orphanet J Rare Dis. (2023) 18(1):329. 10.1186/s13023-023-02879-z 37858231 PMC10585812

[24] Sierra T Crevillen AG Escarpa A. Electrochemical sensor for the assessment of carbohydrate deficient transferrin: application to diagnosis of congenital disorders of glycosilation. Biosens Bioelectron. (2021) 179:113098. 10.1016/j.bios.2021.113098 33636501

[25] Shimada S Ng BG White AL Nickander KK Turgeon C Liedtke KL Clinical, biochemical and genetic characteristics of MOGS-CDG: a rare congenital disorder of glycosylation. J Med Genet. (2022):jmedgenet-2021-108177. 10.1136/jmedgenet-2021-108177 35790351 PMC9813274

