# Supplementary material for: Novel SSR4 gene splice variant leads to congenital disorder of glycosylation, type Iy
Source: Front Pediatr. 2025 Oct 24;13:1651524. doi: 10.3389/fped.2025.1651524 (PMC12592166; doi:10.3389/fped.2025.1651524)
Supplement: Supplementary file 1 [file Datasheet1.zip › Supplementary Figure.pptx]

## Slide 1
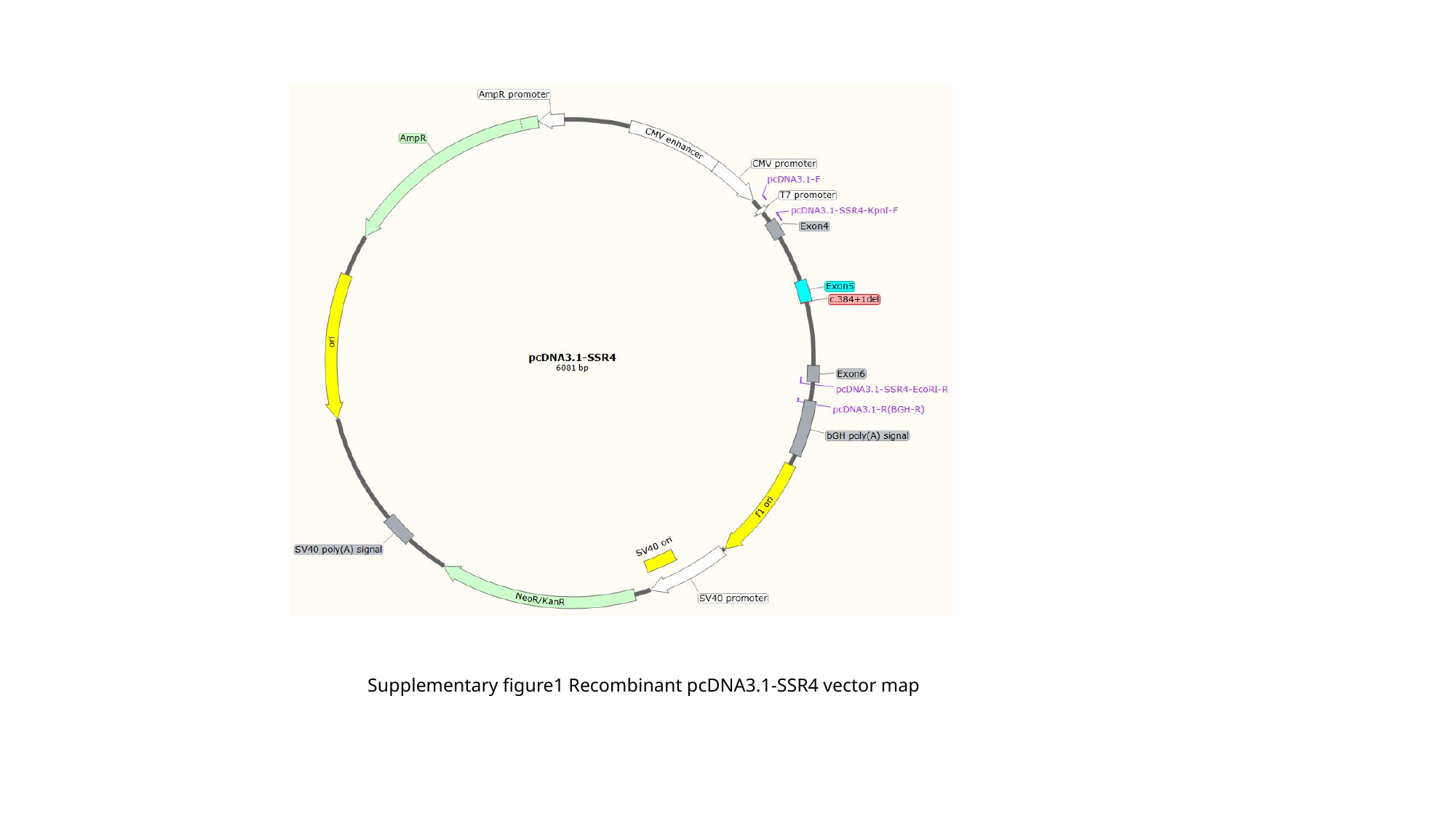

Supplementary figure1 Recombinant pcDNA3.1-SSR4 vector map

## Slide 2
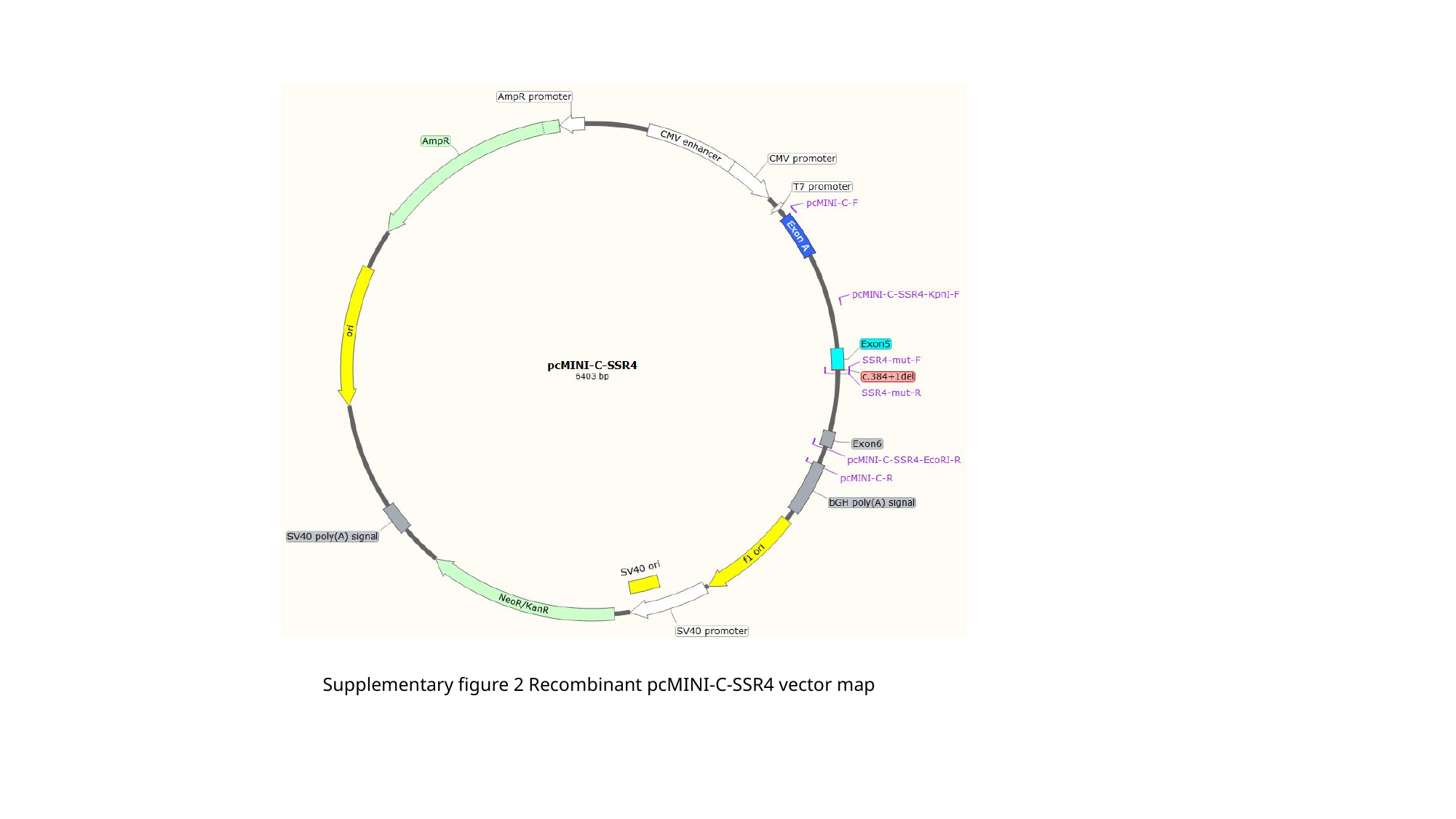

Supplementary figure 2 Recombinant pcMINI-C-SSR4 vector map
